# Visual assessment of right ventricular function by echocardiography: how good are we?

**DOI:** 10.1007/s10554-019-01653-2

**Published:** 2019-06-24

**Authors:** Matthias Schneider, Hong Ran, Stefan Aschauer, Christina Binder, Julia Mascherbauer, Irene Lang, Christian Hengstenberg, Georg Goliasch, Thomas Binder

**Affiliations:** 1 Department of Internal Medicine II, Medical University of Vienna, Waehringer Guertel 18-20, 1090 Wien, Austria; 2 Department of Echocardiography, Nanjing First Hospital, Nanjing Medical University, Nanjing, China

**Keywords:** Transthoracic echocardiography, Right ventricular function, Visual assessment, Eyeballing

## Abstract

The complex anatomy and physiology of the right ventricle (RV) is a major limitation of visual echocardiographic gradation of RV systolic function (RVF). The aim of this study was to compare visual assessment (“eyeballing”) of RVF with gold standard magnetic resonance imaging (MRI)-derived right ventricular ejection fraction (RVEF). Medical professionals from a range of clinical settings and with varying degrees of echocardiography experience were recruited via an online ultrasound teaching platform. In an anonymized web-based test, participants graded RVF in 10 patients with varying degrees of RVF via “eyeballing” of an RV-focused four-chamber view. Two skills were evaluated: (1) ability to differentiate between normal and reduced RVF; and (2) ability to determine the correct degree of RV systolic dysfunction. A total of 868 participants from 99 countries were included. For detection of reduced RVF (MRI-RVEF < 50%), sensitivity was 97.1%, 96,8%, 96.5%, and 95.8% and specificity was 55.7%, 52.8%, 54.6%, and 42.5% for the expert, advanced, intermediate, and beginner groups, respectively. For determination of the correct degree of RV dysfunction, even experienced examiners assigned a diagnosis that was discordant with MRI in > 40% of cases. In the present cohort, “eyeballing” was associated with excellent sensitivity but poor specificity in terms of differentiation between normal and abnormal RVF. Even among experts, classification of the degree of RV dysfunction was imprecise. In accordance with current guidelines, the present data suggest that “eyeballing” should be combined with evaluation of other echocardiographic parameters of RVF.

## Background

Evaluation of right ventricular function (RVF) is an essential component of echocardiographic evaluation. While the geometry of the left ventricle allows precise evaluation of ejection fraction via visual assessment (“eyeballing”) [1, 2], determination of the degree of RVF is rendered challenging by the complex anatomy and physiology of the right ventricle (RV).

Current guidelines for echocardiographic chamber quantification recommend that RVF should be evaluated by both visual assessment and measurement of at least one additional RVF parameter, such as tricuspid annular plane systolic excursion (TAPSE), tissue Doppler imaging of the basal free lateral wall of the RV (S’), or fractional area change (FAC) [3, 4]. These conventional echocardiographic parameters show good correlation with the corresponding cardiac magnetic resonance imaging (CMR) measurements [5]. However, for many of these parameters, echocardiographic evaluation is complex and time consuming. Unsurprisingly, a recent study by the present authors showed that “eyeballing” is the most widely used method for the echocardiographic classification of RVF [6].

Previous studies have investigated the reliability of RVF assessment using “eyeballing” alone. However, the results have been inconsistent. While two studies suggested that “eyeballing” alone is insufficient in terms of determining the degree of RVF [7, 8], a recent study concluded that RVF can be determined reliably by “eyeballing” alone if the examiner is very experienced [9]. These previous studies were performed at single centers and included small numbers of examiners and patients. International data on the use of “eyeballing” alone to assess RVF are therefore lacking.

In recent years, rapid developments in ultrasound technology and the increasingly widespread availability of wireless handheld devices have rendered RV imaging possibilities beyond the context of highly specialized cardiology centers. In the non-cardiology setting, emergency cardiac ultrasound protocols include a quick visual assessment of right and left ventricular function. Since further dissemination of emergency cardiac ultrasound is anticipated, in the foreseeable future RVF will be assessed by more, and inevitably less experienced, examiners. Evaluation of the accuracy of “eyeballing” is therefore crucial in terms of patient safety.

The aims of the present study were twofold. First, to evaluate the ability to differentiate between normal and reduced RVF by “eyeballing”. Second, to assess how visual gradation into class of dysfunction compares to the gold standard CMR-derived right ventricular ejection fraction (RVEF).

## Methods

Participants were recruited via the network of the English-language based online ultrasound teaching platform 123 sonography (https://www.123sonography.com). All participants completed an online questionnaire and a web-based test. The study was open for participation between April 1 and July 31, 2017. The study was approved by the ethics committee of the University of Vienna (EK #1288/2016). The study protocol conformed to the ethical guidelines of the Declaration of Helsinki.

### Questionnaire

No data were collected concerning the names of the participants or their respective institutions. Baseline demographic data were collected, such as age, country in which the participant was employed, profession, and work setting (e.g., university hospital, private practice). To establish study groups, the participants were asked to specify their level of echocardiography experience: beginner, intermediate, advanced, or expert.

### Web-based test

The RV focused apical four-chamber view video loop of 10 real patients was presented online to the study participants. The participants were instructed to grade each RV solely by “eyeballing” according to a four-grade scale (normal, mildly reduced RVF, moderately reduced RVF, severely reduced RVF).

### Selection of patient data

The ten patients were selected retrospectively from the echocardiography database of the University Hospital of Vienna. Inclusion criteria were good echocardiographic image quality and a CMR within three months of the echocardiographic examination. Selected patients represented the entire RVF spectrum from normal to severely reduced systolic function. The clinical, CMR, and echocardiographic characteristics of all 10 patients are summarized in Table 1.

### Echocardiography

**Fig. 1:**
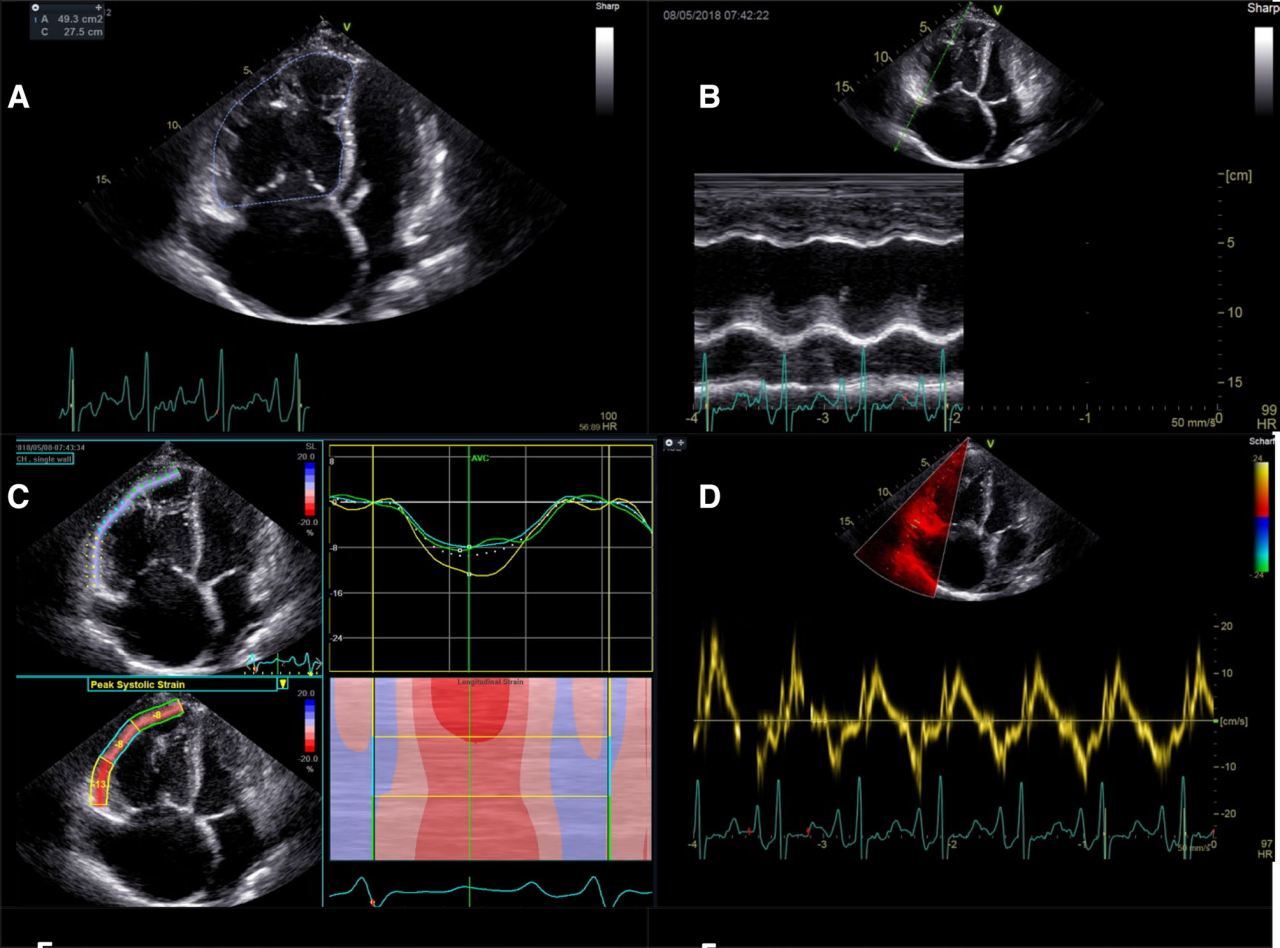
Evaluation of right ventricular function: RV end-diastolic for calculation of right ventricular fractional area change (**a**, RVF reduced if < 35%), TAPSE (**b**, RVF reduced if < 17 mm), longitudinal strain of the free lateral wall of the right ventricle (**c**, RVF reduced if > −20%), and S’ (**d**, RVF reduced if < 0.095 m/s) (1)

Standard transthoracic echocardiograms (2D, Doppler) were obtained from all 10 patients using echocardiography systems equipped with 3.5 MHz transducers (Vivid E9; General Electric Healthcare). Echocardiography was performed in accordance with the recommendations of the American Society of Echocardiography and the European Association of Cardiovascular Imaging [3, 4]. From the stored echo loops of each patient, a high-quality RV-focused apical four-chamber view was selected. In all 10 patients, the following measures were obtained: TAPSE; FAC; global longitudinal strain of the free lateral wall of the RV (GLS-RV); and S’. Figure 1 shows the cut-offs that were used to define reduced RVF [4].

### Cardiac magnetic resonance imaging

All cardiac CMR examinations were performed by using a 1.5-T imager (Magnetom Avanto; Siemens Medical Solutions, Erlangen, Germany) with standard protocols. Steady-state images were used for cine imaging (repetition time msec/echo time msec, 3.2/1.2; ip angle, 64°; voxel size, 1.431.436 mm; 1,803,256 matrix). A transaxial stack of images was used for volumetric assessment of RVs. Trabeculations, papillary muscles, and the right ventricular outflow tract were included as part of the RV volume as previously suggested [10]. RV dysfunction was defined as RV ejection fraction < 50% and was further subdivided into mildly decreased (RV ejection fraction 40–49%), moderately decreased (RV ejection fraction 30–39%) and severely decreased (RV ejection fraction < 30%) [11]. Two independent observers (SA, JM) blinded to clinical data read all CMR studies.

All patients had undergone both CMR and an echocardiographic examination within a mean period of 12 ± 22 days. According to CMR, five (50%) patients had normal RVEF (RVEF > 50%), one (10%) patient had mildly reduced RVEF (RVEF: 40–50%), three (30%) patients had moderately reduced RVEF (RVEF: 30–40%), and one (10%) patient had severely reduced RVEF (RVEF < 30%). Median RVEF was 52% (range 21–74%). Detailed information on CMR assessment is presented in Table 1.

**Table 1:** Clinical and CMR data of the ten patients evaluated by the survey participants

Patient	Cardiac diagnosis	RVEF (CMR, %)	TAPSE (mm)	S’ (m/s)	FAC (%)	GLS-RV (%)
1	Heart failure with preserved ejection fraction	74	30	0.21	60	− 40.33
2	Coronary artery disease	71	20	0.10	48	− 25.33
3	TTR-amyloidosis, postcapillary pulmonary hypertension	65	17	0.10	50	− 26.67
4	Dilated cardiomyopathy	58	22	0.14	47	− 24.33
5	Sudden cardiac death survivor	55	27	0.16	60	− 31
6	Severe mitral regurgitation, postcapillary pulmonary hypertension	49	16	0.11	39	− 23
7	Idiopathic pulmonary arterial hypertension	38	16	0.13	27	− 19.33
8	Tachy-cardiomyopathy	38	14	0.09	39	− 16.33
9	Combined pre/postcapillary pulmonary hypertension	34	13	0.09	30	− 14.33
10	Idiopathic pulmonary arterial hypertension	21	10	0.10	22	− 5

*RVEF* right ventricular ejection fraction, *CMR* cardiac magnetic resonance imaging, *TAPSE* tricuspid annular plane systolic excursion, *S’* tissue Doppler imaging basal lateral segment of the free lateral wall of the right ventricle, *FAC* fractional area change, *GLS-RV* global longitudinal strain of the free lateral wall of the right ventricle

### Statistical analysis

Categorical data are presented as absolute numbers and percentages. Inter-group comparisons of descriptive data were performed using the *χ*^2^ test or Fisher’s exact test, as appropriate. A p-value of < 0.05 was considered statistically significant. All statistical analyses were performed using SPSS Version 24 (IBM SPSS, USA).

First, the ability to detect a decrease in RVF (CMR-RVEF < 50%) via “eyeballing” alone was evaluated. Sensitivity, specificity, negative predictive value, positive predictive value, area under the curve (AUC), and accuracy were calculated.

Apart from correct classification into normal or decreased function, consensus between a large group of examiners in terms of the degree of dysfunction is necessary to enable uniform nomenclature and reliable follow-up examinations. Therefore, a scoring was performed for concordance between visual assessment of the degree of RV systolic dysfunction and the CMR gold standard (0 points, concordant; 1 point, 1 grade difference; 2 points, 2 grade difference; 3 points, 3 grade difference). For instance, if CMR-RVEF was normal with 55%, and the participant graded the RV as mildly reduced, a score of 1 point was attributed. For each participant, the total score for the evaluation of all 10 patients was calculated. The lower the score, the better the agreement between the participant and the CMR diagnosis.

We compared two different systems of classification. A three-grade scale (normal, reduced, severely reduced RVF) and a four-grade scale (normal, mildly reduced, moderately reduced, severely reduced RVF). For the three-grade scale the participants’ ratings for mildly and moderately reduced RVF were counted as one combined category (reduced RVF).

## Results

### Demographic and professional characteristics of the study participants

**Fig. 2:**
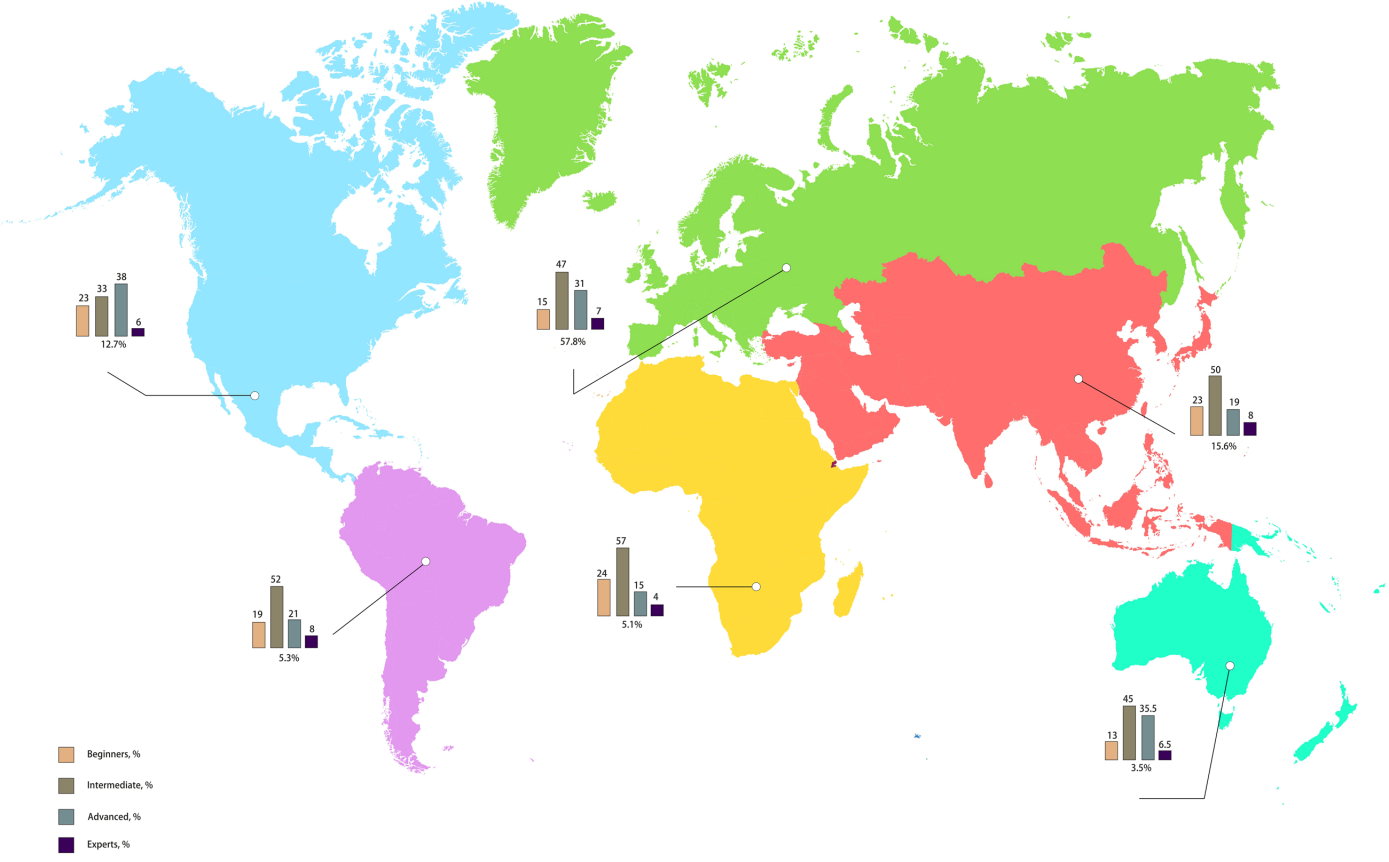
Regional distribution of the study's participants and their respective level of expertise

A total of 868 participants completed both the questionnaire and the web-based test. Eighty-one percent of the cohort were between 30 and 60 years of age. A total of 40% were cardiologists; 22% were sonographers; and 20% were internists. Employment settings comprised the following: university hospitals, 29%; non-tertiary hospitals, 47%; and private practice, 21%. The number of participants in each of the four study groups was as follows: 17%, beginners; 47%, intermediate; 29%, advanced; and 7%, expert. The participants were based in 99 different countries, including the following: the United States of America, 11%; Germany, 8%; and Austria, 6%. The demographic and professional characteristics of the participants are shown in Table 2 and Fig. 2.

**Table 2:** Demographic and professional characteristics of the study participants (n = 868)

Characteristics	All	Medical doctors	Sonographers
Number of participants, n (%)	868 (100)	675 (78)	193 (22)
Age (years)			
< 30, n (%)	75 (9)	48 (7)	27 (14)
30–39, n (%)	309 (36)	253 (38)	56 (29)
40–49, n (%)	239 (27)	188 (28)	51 (26)
50–59, n (%)	156 (18)	118 (17)	38 (20)
60–69, n (%)	81 (9)	63 (9)	18 (9)
> 69, n (%)	8 (1)	5 (1)	3 (2)
Work setting			
University hospital, n (%)	251 (29)	210 (31)	41 (21)
Hospital, n (%)	407 (47)	305 (45)	102 (53)
Private practice, n (%)	186 (21)	143 (21)	43 (22)
Other, n (%)	24 (3)	17 (3)	7 (4)
Level of expertise			
Beginner, n (%)	144 (17)	117 (17)	27 (14)
Intermediate, n (%)	410 (47)	333 (49)	77 (40)
Advanced, n (%)	255 (29)	183 (27)	72 (37)
Expert, n (%)	59 (7)	42 (6)	17 (9)

#### Detection of reduced RVF (CMR-RVEF < 50%)

Correct detection of reduced RVF (CMR-derived RVEF < 50%) was analyzed for each of the four study groups. Sensitivity was 95.8%, 96.5%, 96,8%, and 97.1% for the beginner, intermediate, advanced, and expert groups, respectively. Specificity in these four groups was 42.5%, 54.6%, 52.8%, and 55.7%, respectively. Better concordance was found for the intermediate, advanced, and expert groups than for beginners (Table 3). When beginners were compared with the combined advanced, moderately advanced, and expert groups, the area under the curve (AUC) was 0.69 for beginners vs. 0.75 in the combined non-beginners group.

**Table 3:** Diagnostic accuracy for detection of reduced right ventricular function (mild, moderate, or severe) using CMR-RVEF > 50% as the gold standard for normal RVF

	Sensitivity (95% CI)	Specificity (95% CI)	PPV	NPV	Accuracy
Beginner	95.8 (94.1–97)	42.5 (39.1–46)	62.5 (61–63.9)	90.9 (87.7–93.4)	69.1 (66.8–71.4)
Intermediate	96.5 (95.6–97.3)	54.6 (52.4–56.7)	68 (66.9–69)	94 (92.6–95.2)	75.5 (74.2–76.8)
Advanced	96.8 (95.7–97.7)	52.8 (50–55.5)	67.2 (65.9–68.5)	94.3 (92.5–95.8)	74.8 (73.1–76.5)
Experts	97.1 (94.5–98.6)	55.7 (50–61.4)	68.7 (65.9–71.4)	95 (90.8–97.3)	76.4 (72.8–79.7)

*CMR* cardiac magnetic resonance imaging, *RVF* right ventricular function, *PPV *positive predictive value, *NPV* negative predictive value

**Fig. 3:**
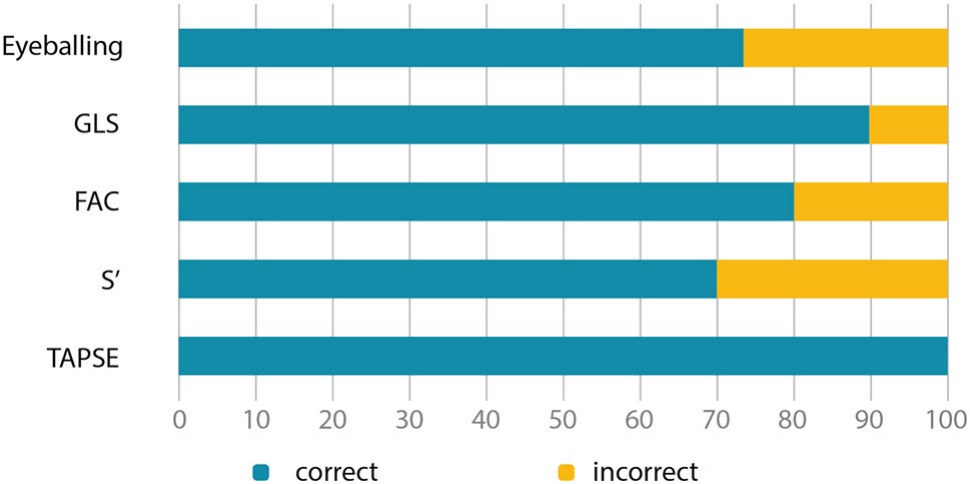
Correct identification of reduced right ventricular function (RVF, defined as cardiac magnetic resonance imaging derived right ventricular ejection fraction <50%) of the different methods of RVF gradation. *GLS* global longitudinal strain, *FAC* fractional area change, *S’* tissue Doppler imaging basal free lateral wall of the right ventricle, *TAPSE* tricuspid annular plane systolic excursion

Correct identification of reduced RVF was compared between “eyeballing” and echocardiographic parameters. While the study participants differentiated correctly in 74% of cases by “eyeballing”, correct differentiation was found for TAPSE in all 10 patients, GLS-RV in nine patients, FAC in eight patients, and S’ in seven patients (Fig. 3).

#### Determination of the degree of RV systolic dysfunction

**Fig. 4:**
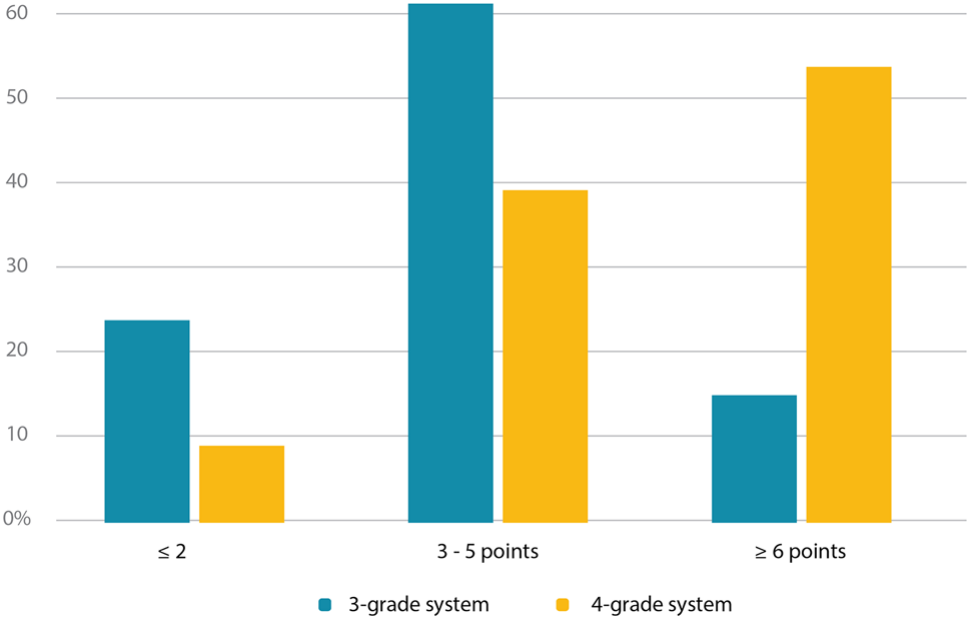
Comparison of 3-grade system (normal, reduced, severely reduced) against 4-grade system (normal, mildly reduced, moderately reduced, severely reduced). ≤ 2 points indicate excellent concordance with CMR, ≥ 6 points indicate poor concordance with CMR. *CMR* cardiac magnetic resonance imaging

Two different systems of systolic function gradation were compared. For the four-grade system, 8% of the participants scored ≤ 2 points, indicating very good concordance with CMR, and 54% of the participants scored ≥ 6 points, indicating poor concordance with CMR. In the three-grade system, 23% of the participants scored ≤ 2 points, and 14% of the participants scored ≥ 6 points (Fig. 4).

#### Accuracy in determining the degree of RV systolic dysfunction

**Fig. 5:**
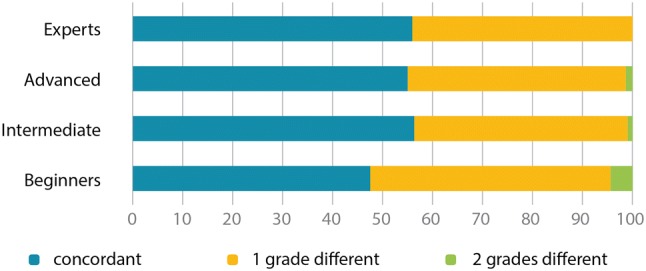
Concordance with cardiac magnet resonance imaging derived right ventricular ejection fraction of the different levels of expertise

Further analysis was then performed for the three-grade system. Participants from the expert, advanced, and intermediate groups assigned a diagnosis that was concordant with CMR in 56%, 56%, and 57% of cases, respectively. Participants from these groups assigned a level that was one grade different to CMR in 43%, 43%, and 42% of cases, respectively, and two grades different to CMR in 0.2%, 1%, and 1% of cases, respectively. Participants from the beginners group were concordant with CMR in 48% of cases, and assigned a level that was one grade different to CMR in 48% of cases, and two grades different to CMR in 4% of cases (Fig. 5). Less than 10% of the 868 participants assigned the correct diagnosis in 100% of cases. A total of 80% or more correct diagnoses were assigned by the following percentages of individuals from each study group: 33%, beginners; 51%, intermediate; 47%, advanced; and 55%, expert. Compared with the other three groups, significantly fewer correct diagnoses, and more incorrect diagnoses, were observed in the beginners group (p < 0.001).

## Discussion

The present investigation of “eyeballing” alone for the evaluation of RVF showed that, while sensitivity for the detection of reduced RVF was excellent in all expertise levels, including beginners, specificity was poor. In addition, determination of the degree of RV systolic dysfunction was imprecise.

To our knowledge, the present study is the first to utilize the potential of the internet in order to investigate this issue in an international context. A major strength of the analyses was the large study cohort, which represented a broad range of echocardiography expertise and clinical settings.

### Detection of reduced RVF (CMR-RVEF < 50%)

Evaluation of the ability to detect reduced RVF revealed excellent sensitivity and negative predictive value in all four study groups. The more experienced the examiner, the better were sensitivity, specificity, and accuracy. Differences between the groups were marginal, though. Even beginners identified reduced RVF in most cases. This is reassuring, since reduced RVF is a crucial finding that must prompt further diagnostic workup. For instance, reduced RVF impacts the management of pulmonary embolism. In these patients, echocardiography is frequently performed by inexperienced non-cardiologists, and occasionally with the use of wireless handheld devices, for example, in the emergency medicine setting. In these cases, measurement of more sophisticated RVF parameters is precluded. The present data indicate that echocardiography is a very useful tool, also for non-cardiologists in non-tertiary settings. Thus, in view of the ever decreasing cost and increased availability of ultrasound machines, this suggests that basic medical education should involve expanded training in basic echocardiography.

Specificity was very poor in all four study groups, with the highest specificity being found among experts and the lowest among beginners. The low specificity observed in the present analyses may have been attributable to the fear of overlooking patients with reduced RVF.

Analyses were also performed to determine the degree to which reduced RVF could be detected using more sophisticated echocardiography parameters. TAPSE reliably distinguished between normal and reduced RVF in all 10 patients, while both FAC and GLS-RV were more accurate than “eyeballing” alone. Therefore, comprehensive evaluation of RVF should include a range of parameters to correct for potential visual errors. In cases of diagnostic doubt, the present data suggest that an experienced examiner should re-evaluate the acquired images, and that other imaging modalities, such as CMR, should be considered.

### Accuracy of the determination of the degree of RV systolic dysfunction

Across all four study groups, significant disagreement was apparent, in particular with respect to mildly and moderately reduced RVF. For this reason, accuracy with the three-grade scale was superior. If diagnosis is based solely on visual assessment and 2D echo parameters such as TAPSE, S’, FAC, and GLS-RV, the present authors propose the use of a three-grade system for determination of the degree of RV systolic dysfunction. With 3D echocardiography, an additional parameter has become available in recent years. If a reliable 3D echocardiography dataset is available, RVEF can be calculated, thus allowing further classification into mildly and moderately reduced RVF.

When applying the three-grade system, even in the expert group, only half of the examiners classified 80% or more of the patients in accordance with gold standard CMR values. While beginners had slightly lower rates of concordant diagnoses, similar results were found across the expert, advanced, and intermediate groups. These findings suggest that this skill can be learned quickly and is performed on a similar, albeit unsatisfactory, level across all levels of expertise beyond novice status.

## Limitations

The present study had limitations. First, participants were recruited from the users of an online teaching platform, which may have introduced a selection bias. This selection bias may have led to the inclusion of professionals with an interest in continued medical education and an open attitude towards new technologies. Level of expertise was self-reported, allowing for false classification both to more and to less advanced levels than accurate. Second, evaluation of RVF was based solely on a RV-focused four-chamber view. In clinical practice, RVF should always be evaluated using several angulations of the RV. However, the one-loop questions enabled a very low threshold for participation and thus a large study cohort. Third, in the real-world setting, assessment of RVF is also dependent on the ability to acquire good images. All video loops in the present study had good image quality. This precludes generalizability of the study. Fourth, as suggested by the literature, CMR-RVEF was used as the gold standard for RVF. Ejection fraction describes volume change during the cardiac cycle. In the RV, a close relationship exists between myocardial contraction, pulmonary pressure, and the pulmonary vasculature, which is not accounted for by RVEF. Future research is necessary to determine whether superior parameters for gold standard RVF measurement are available, which might correlate better with clinical status and outcome. Finally, CMR and echo were not acquired simultaneously. However, the mean time interval between the two examinations was only 12 days, and all patients were in stable clinical condition with no relevant volume shifts having been recorded between the two assessments.

## Conclusion

To our knowledge, the present study is the first international investigation to demonstrate that “eyeballing” alone differentiates between normal and abnormal RVF with excellent sensitivity but poor specificity, and that accurate classification of the degree of RV systolic dysfunction via “eyeballing” alone is imprecise, even among expert echocardiographers. Better concordance with the gold standard was found for the three advanced groups as compared with beginners. However, overall assessment quality was unsatisfactory. In accordance with current guidelines, the present data suggest that “eyeballing” should be combined with measurement of other parameters of RVF.
